# Replication of Association between *ADAM33* Polymorphisms and Psoriasis

**DOI:** 10.1371/journal.pone.0002448

**Published:** 2008-06-18

**Authors:** Valérie Siroux, Emmanuelle Bouzigon, Marie-Hélène Dizier, Isabelle Pin, Florence Demenais, Francine Kauffmann, for the EGEA cooperative group

**Affiliations:** 1 Inserm, U823, Centre de Recherche Albert Bonniot, Epidémiologie des cancers et des affections graves, La Tronche, France; 2 Univ Joseph Fourier, Grenoble, France; 3 INSERM, U794, Fondation Jean Dausset/CEPH, Paris, France; 4 Univ Evry, Evry, France; 5 INSERM, U535, Villejuif, France; 6 Univ Paris-Sud, IFR69, Villejuif, France; 7 CHU Grenoble, Pédiatrie, Grenoble, France; 8 Inserm, U780, Epidemiology and Biostatistics, Villejuif, France; The University of Queensland, Australia

## Abstract

Polymorphisms in *ADAM33*, the first gene identified in asthma by positional cloning, have been recently associated with psoriasis. No replication study of this association has been published so far. Data available in the French EGEA study (Epidemiological study on Genetics and Environment of Asthma, bronchial hyperresponsivensess and Atopy) give the opportunity to attempt to replicate the association between *ADAM33* and psoriasis in 2002 individuals. Psoriasis (n = 150) has been assessed by questionnaire administered by an interviewer and a sub-sample of subjects with early-onset psoriasis (n = 74) has been identified based on the age of the subjects at time of interview (<40 years). Nine SNPs in *ADAM33* and 11 SNPs in *PSORS1* were genotyped. Association analysis was conducted by using two methods, GEE regression-based method and a likelihood-based method (LAMP program). The rs512625 SNP in *ADAM33* was found associated with psoriasis at p = 0.01, the usual threshold required for replication (OR [95% CI] for heterozygotes compared to the reference group of homozygotes for the most frequent allele = 0.61 [0.42;0.89]). The rs628977 SNP, which was not in linkage disequilibrium with rs512625, was significantly associated with early-onset psoriasis (p = 0.01, OR [95% CI] for homozygotes for the minor allele compared to the reference group = 2.52 [1.31;4.86]). Adjustment for age, sex, asthma and a *PSORS1* SNP associated with psoriasis in the EGEA data did not change the significance of these associations. This suggests independent effects of *ADAM33* and *PSORS1* on psoriasis. This is the first study that replicates an association between genetic variants in *ADAM33* and psoriasis. Interestingly, the 2 *ADAM33* SNPs associated with psoriasis in the present analysis were part of the 3-SNPs haplotypes showing the strongest associations in the initial study. The identification of a pleiotropic effect of *ADAM33* on asthma and psoriasis may contribute to the understanding of these common immune-mediated diseases.

## Introduction

Psoriasis is an inflammatory disease of the skin, phenotypically heterogeneous, characterized by hyperproliferation and remodeling, often expressed by chronic plaque-type [1], [2]. It is a multifactorial disease, with a genetic basis and with environmental factors involved in exacerbating or in triggering the disease [3]. Strongly associated with the major histocompatibility complex, especially for early-onset forms, this auto immune disease exhibits a Th1 dominant cytokine profile, with an important role of various interferon responses [4]. Environmental triggers include bacterial skin infections and tissue injury. Its association with other disorders, such as Crohn's disease, arthritis, diabetes and coronary heart disease [5] support the hypothesis of common genes with other diseases. Multiple genome-wide linkage analyses and genetic association analyses have been performed in order to elucidate the genetic component of psoriasis. There is still limited evidence of the implication of other genes than *PSORS1*, located in the MHC region [6], but the role of several other genes has been suggested [7], [8]. Recently a genome-wide scan in French extended families of psoriasis confirmed the presence of a psoriasis susceptibility locus on chromosome 20p13 [9], and identified by positional cloning the *ADAM33* gene (A Disintegrin And Metalloproteinase 33) as a psoriasis susceptibility gene [10]. *ADAM33* was the first gene identified in asthma by positional cloning [11]. Its function is not well understood, but has a putative role in airway remodelling [12]. The association of *ADAM33* with asthma has been confirmed in some studies, but not all. No replication study on the association between genetic variations in *ADAM33* and psoriasis has been published so far.

The aim of the present paper was to attempt to replicate the association between polymorphisms in the *ADAM33* gene and psoriasis, taking advantage of data collected in the French Epidemiological study on the Genetics and environment of Asthma (EGEA), a study in which no association of *ADAM33* with asthma was observed [13].

## Results

Among the 2002 subjects included in the present analysis, 150 had psoriasis (7.5%) and half of the subjects with psoriasis had early-onset psoriasis (n = 74). Subjects with psoriasis were older than those without psoriasis (Table 1).

**Table 1:** Description of the population.

	No psoriasis, n = 1852	Psoriasis, all n = 150	Psoriasis with early-onset (<40 years), n = 74	p* between all psoriasis and no psoriasis
Sex, % males	51.1	52.7	50.0	0.73
Age, m±sd	38.8±17.9	44.6±15.9	32.2±11.3	0.0002
Age, <40 years %	52.2	34.7		<0.0001
Smoking:				
Never smokers, %	50.7	40.7	45.7	0.07
Ex-smokers, %	24.9	27.6	20.0	
Current smokers, %	24.4	31.7	34.3	
Asthma, %	44.3	36.7	37.8	0.05

* p value assessed using GEE model.

In the single-SNP analysis and considering a general codominant model for each SNP effect, one SNP, rs512625, showed association with psoriasis that reached the 1% significance level classically required for replication (Table 2). Heterozygotes for this SNP had a decreased risk for psoriasis as compared to the reference group of homozygotes for the most frequent allele (OR = 0.61 [0.42; 0.89]) (Table 3). Moreover, a dominant model was rejected against the general codominant model (p = 0.02). When examining early-onset psoriasis, we found association with another SNP, rs628977, at the 1% level (Table 2). Homozygotes for the minor T allele had a higher risk for early-onset psoriasis as compared to the reference group (OR = 2.52 [1.31; 4.86]) (Table 3). A recessive model for rs628977 (which fits the data, p = 0.55) increased the significance (p = 0.003). Two polymorphisms in the PSORS1 gene, rs3131000 and rs3130559, were associated with psoriasis (p<0.01; Supplementary, Table S1). None of these two SNPs was significantly associated with early-onset psoriasis.

**Table 2:** Test of association of psoriasis and early-onset psoriasis with *ADAM33* and *PSORS1* SNPs under a general genetic model using the GEE method.

SNP	Location (bp)	Nucleotide change Ref. allele>alt. allele	MAF*	Psoriasis p value**	Early-onset psoriasis p value**
**ADAM33**					
rs6084432	3583653	G>A	0,15	0.80	0.15
rs512625	3588378	G>A	0,29	**0.01**	0.06
rs677044	3589431	A>G	0.23	0.33	0.15
rs543749	3589679	G>T	0.12	0.83	0.41
rs628977	3589721	C>T	0.38	0.10	**0.01**
rs2787095	3595943	G>C	0.38	0.69	0.06
rs554743	3602142	C>T	0.27	0.32	0.37
rs2853215	3606255	G>A	0.27	0.79	0.07
rs4815596	3611800	G>A	0,13	0.73	0.98
**PSORS1**					
rs2284177	31197574	G>C	0,21	0.69	0.72
rs3131000	31199252	G>C	0,41	**0.03**	0.12
rs3094207	31199388	C>G	0,36	0.07	0.08
rs3094205	31199841	A>G	0,36	0.08	0.10
rs3095307	31200028	G>C	0,44	0.79	0.72
rs3094198	31201306	A>G	0,48	0.58	0.60
rs3130558	31205162	C>G	0,25	0.18	0.56
rs3130559	31205280	C>T	0,22	**0.01**	0.47
rs3130560	31205432	T>G	0,26	0.26	0.63
rs3823418	31208921	G>A	0,21	0.86	0.43
rs2074478	31213612	C>T	0,17	0.99	0.97

* Minor Allele Frequency.

** p value for a general genetic model (2df).

**Table 3:** Association between psoriasis and two genetic variations (rs512625 and rs628977) in ADAM33.

	Genotype	Non psoriasis n = 1852	Psoriasis, all n = 150	Early-onset psoriasis, n = 74	OR [95% CI] associated with psoriasis	p value	OR [95% CI] associated with early-onset psoriasis	p value
**Single SNP analyses**								
**rs512625**								
- Univariate analysis	GG	50.0	58.1	60.3	1.00	**	1.00	
	GA	41.2	29.1	27.4	0.61 [0.42; 0.89]	0.01	0.57 [0.34; 0.97]	0.04
	AA	8.8	12.8	12.3	1.28 [0.72; 2.30]	0.40	1.23 [0.57; 2.64]	0.60
- Adjusted for age, sex, asthma and *PSORS1* rs3131000	GG				1.00	**	1.00	*
	GA				0.62 [0.43;0.91]	0.015	0.57 [0.33;0.97]	0.04
	AA				1.33 [0.74;2.41]	0.34	1.19 [0.55;2.58]	0.65
**rs628977**								
- Univariate analysis	CC	38.5	33.6	29.8	1.00		1.00	**
	CT	47.1	45.3	43.3	1.11 [0.76; 1.63]	0.59	1.18 [0.68; 2.03]	0.55
	TT	14.4	21.2	26.9	1.72 [1.03; 2.86]	0.04	2.52 [1.31; 4.86]	0.006
- Adjusted for age, sex, asthma and *PSORS1* rs3131000	CC				1.00		1.00	***
	CT				1.11 [0.76;1.63]	0.59	1.15 [0.67;2.00]	0.61
	TT				1.62 [0.97;2.72]	0.07	2.59 [1.35;4.95]	0.004
**2-SNPs analysis**								
rs512625	GG				1.00	**	1.00	**
	GA				0.59 [0.40; 0.88]	0.01	0.51 [0.28; 0.90]	0.02
	AA				1.08 [0.58; 2.00]	0.81	0.94 [0.42; 2.09]	0.88
rs628977	CC				1.00		1.00	***
	CT				1.20 [0.81; 1.78]	0.36	1.38 [0.80; 2.41]	0.25
	TT				1.84 [1.08; 3.13]	0.03	2.93 [1.47; 5.84]	0.002

p value for the global two-degrees of freedom test under the general codominant model: ^*^ p<0.10; ^**^p<0.05, ^***^ p<0.01.

The effects on psoriasis of the two *ADAM33* SNPs found associated with the disease in the univariate analysis were then tested by multivariate analysis adjusting for the effects of age, sex, asthma and rs3131000, one of the most strongly psoriasis-associated *PSORS1* SNP in our data (Table 3). Association of rs512625 with psoriasis and the OR estimate in heterozygotes remained unchanged as compared to the univariate analysis (Table 3). Similarly, rs629877 remained associated with early-onset psoriasis with homozygotes for the T allele showing an increased risk (p = 0.004) (table 3). Moreover, repeating the multivariate analysis by adjusting for the other *PSORS1* SNP associated with psoriasis (rs3130559) led to the same results (data not shown).

Since low linkage disequilibrium (LD) was observed between rs628977 and rs512625 (LD coefficient D′ = 0.34; see online supplement Table S2), we conducted a two-SNP analysis for both psoriasis and early-onset psoriasis by including these two polymorphisms in the regression model. This 2-SNP analysis showed that these polymorphisms were jointly associated to both psoriasis and early-onset psoriasis and had statistically independent effects, these effects being of the same order of magnitude as in the single-SNP analyses (Table 3). A stratified analysis on rs512625 genotypes indicated an excess risk for psoriasis in TT homozygotes for rs628977 that was similar for each rs512625 genotypic class (results not shown). This suggests there is no interaction between these 2 SNPs.

Analyses were also repeated with a recently proposed likelihood-based method that can take into account the whole family structures and conditions upon the observed affection status, as implemented in the LAMP program [14], [15]. This approach confirmed the association between rs512625 and psoriasis (p = 0.007 for the 2 df likelihood-ratio test under a general codominant model for the SNP effect) with a protective effect in heterozygotes as found before with the regression approach (ratio of penetrances of GA *versus* GG = 0.62). This method also showed an association between rs628977 and early-onset psoriasis (p = 0.03 for the 2 df test) with an increased risk in TT homozygotes (ratio of penetrances of TT *versus* CC = 2.23) (results not shown).

## Discussion

For the first time, an association between *ADAM33* and psoriasis initially observed in French families of psoriasis is replicated [10]. Interestingly, rs512625, the *ADAM33* SNP showing the strongest association with psoriasis considered as a whole in the present analysis, was also among the SNPs showing the strongest association with psoriasis by single SNP and haplotype analyses in the initial study [10]. Another SNP, rs628977, was specifically associated with early-onset psoriasis in the present analysis. This locus has not been identified by the single-SNP analysis conducted by Lesueur et al. [10], but this SNP was part of the 3-SNPs haplotypes showing the strongest associations with psoriasis. In both studies, the T allele was associated with an increased risk for psoriasis (or early-onset psoriasis, as detected here). Our results also suggest that these two *ADAM33* SNPs have independent effects on psoriasis.

Estimates of the odds-ratios for rs512625 showed a protective effect on psoriasis in heterozygotes but not in homozygotes for the minor allele. This decreased in risk observed in heterozygotes only may result from a more complex underlying model involving interactions between SNPs at a given locus or with other factors. This is in line with the results of the initial study [10] where the most significant association was observed in the 3-SNPs haplotype analysis.

The present analysis has been performed in a large case-control and family study for asthma and asthma-related phenotype (EGEA). In this population, the frequency of psoriasis was 7.5%, higher than the prevalence of psoriasis expected in Caucasian populations (2–4%) [16]. It may be explained by two complementary aspects: 1) The EGEA data, composed of 388 asthmatic cases and their first degree relatives and controls, is not representative of a population-based study; 2) It has been previously shown that questionnaire-based assessment of psoriasis leads to an over-estimate of the prevalence compared to dermatological examination-based assessment of psoriasis [17]. Nevertheless, the significant associations observed between psoriasis and SNPs in *PSORS1*, one locus identified consistently by several linkage and association studies, indicates a good validity of the psoriasis phenotype in the EGEA study.

The sub-sample of early-onset psoriasis has been identified using the age of the subjects at time of interview since age at onset was not recorded in the EGEA study. Consequently, psoriasis patients aged 40 years and older cannot be identified as late-onset psoriasis since they include subjects with both early-onset and late-onset psoriasis. Although it has been previously shown that HLA-cw6 (*PSORS1*) is more specifically associated with an earlier age of onset, 2 *PSORS1* SNPs were found significantly associated with psoriasis considered as a whole in the EGEA study but none of them was significantly associated with early-onset psoriasis. The lack of association between *PSORS1* SNPs and early-onset psoriasis in the EGEA study may result from a lack of power since the ORs observed for rs3131000 genotypes were higher when considering early-onset psoriasis than psoriasis as a whole. Regarding *ADAM33*, the rs628977 SNP was more specifically associated with early-onset psoriasis. As previously observed for HLA-cw6, SNPs in *ADAM33* may also be more specifically associated with early-onset psoriasis.

The analysis conducted allowed to adjust for potential confounders. Interestingly, after adjustment on *PSORS1* polymorphisms, the association between *ADAM33* and psoriasis remained significant. In agreement with results from Lesueur et al, *ADAM33* and *PSORS1* seem to be independently involved in psoriasis.

The function of the *ADAM33* gene is not yet well known, but several arguments support the biological plausibility of an association between psoriasis and *ADAM33*. *ADAM33* is expressed in airway smooth muscle and lung fibroblasts and also in the skin [18]. In parallel, genetic variants in *ADAM33* were found associated with asthma and psoriasis, two diseases characterized by tissue remodelling. This is consistent with a pleiotropic effect of this gene. There is active on-going research regarding *ADAM33* and the functional role of various SNPs starts to be unravelled [19]. Of interest, in the context of psoriasis, a Th1 disease, is the demonstration of a down regulation of the expression of *ADAM33* by IFNG (Interferon-gamma), the prototypical Th1 cytokine, in airway smooth muscle cells [20]. Immunological control in psoriasis is complex and increasing evidence supports that not only Th1, but also Th17, may play a major role in the control of epidermal hyperplasia [21], [22]. The independent association of *PSORS1* with other genes, such as *ADAM33*, is coherent with the role of various processes including adaptative and innate immunity, inflammation and remodelling in the occurrence and perpetuation of psoriasis, a complex disease.

Further research is needed to further replicate the present association, to assess potential associations in various psoriasis subtypes by studying larger samples and to understand the potential regulation of *ADAM33* at the epidermal level. The identification of a pleiotropic effect of *ADAM33* on asthma and psoriasis may contribute to the understanding of these common immune-mediated diseases.

## Materials and Methods

Psoriasis has been assessed by questionnaire administered by an interviewer in more than 2000 subjects included in the EGEA study with a positive answer to ‘Have you ever been treated or followed for psoriasis”. The first EGEA survey (EGEA1) has been conducted from 1991 to 1995 and the protocol and descriptive characteristics have been described elsewhere [23], [24]. Briefly, 388 families of asthmatic cases were recruited in chest clinics of six clinical centers and 415 controls were recruited mostly in electoral rolls and also in surgery departments and a check-up center. One of the first genome screens for asthma has been conducted in EGEA [25]. A twelve year follow-up of the study (EGEA2) has been conducted for 2003 to 2006. Among the alive cohort, 92% (n = 1845) completed a short self-questionnaire and 77% (n = 1543) participated to the second phase of the study. In addition, 58 new family members where included in the study at the second survey. Written consent was obtained from all participants at both surveys. Ethical approval to carry out the study was obtained for both surveys from the relevant committees (Cochin Royal Hospital, Paris for Egea1 and Necker-Enfants Malades Hospital, Paris for Egea2).

As part of a panel of asthma candidate genes, 9 SNPs belonging to *ADAM33* gene were genotyped in 2009 subjects using either Taqman Probes (Applied Biosystems, Foster City, California) on an AB17900HT Sequence Detection System or 1536-plex Illumina GoldenGate assay (Illumina, San Diego, CA) at the Centre National de Génotypage (CNG, Evry, France). Moreover, in the context of HLA region genotyping using Illumina GoldenGate assay, a total of 11 SNPs within *PSORS1* gene were available in our data for 1203 subjects. The consistency of the SNP data with Mendelian inheritance was evaluated using the PedCheck program [26]. Test of Hardy-Weinberg equilibrium was performed using an exact test [27]. No SNP showed Hardy-Weinberg disequilibrium.

The present analysis has been conducted on 2002 individuals with available data on both psoriasis and at least one polymorphism in the *ADAM33* gene.

### Statistical analyses

The association between psoriasis and polymorphisms of *ADAM33* and *PSORS1* genes was assessed using two complementary statistical methods: 1) a logistic regression model taking into account familial dependence of the observations (GEE model. Proc GENMOD using SAS statistical software); 2) a likelihood-based method taking into account the whole family structures and conditioning upon the observed affection status (implemented in LAMP program) [14], [15]. The effect of each SNP on disease (univariate analysis) was tested under a general genetic model (2 degrees of freedom test) and the odds-ratios (ORs) (or ratios of penetrances using LAMP) were calculated using as reference group the homozygotes for the most frequent allele. Multivariate analyses were conducted using the GEE regression approach. These multivariate analyses allowed testing for SNP effect while adjusting for age, sex and asthma, three characteristics significantly associated with psoriasis in the EGEA data, and for genotypes at rs3131000, the *PSORS1* SNP most strongly associated with psoriasis in our data. Multiple regression analysis was also used to test for the effect of multiple *ADAM33* markers on psoriasis.

Since two types of psoriasis have already been widely described according to the age of onset, before or after age 40, we identified a sub-sample of psoriasis with early-onset psoriasis based on the age of the subjects at time of interview (age of onset of psoriasis was not recorded): early-onset psoriasis consisted of the sub sample of individuals younger than 40 years of age when they replied positively to the psoriasis question [3], [28]. However, psoriasis patients older than 40 years of age could not be considered as late-onset psoriasis. Association analyses were thus carried out by examining either psoriasis as a whole or early-onset psoriasis in subjects less than 40 years old.

## Supporting Information

Table S1 (0.04 MB DOC)

Table S2 (0.04 MB DOC)
