# Supplementary material for: Replication of Association between ADAM33 Polymorphisms and Psoriasis
Source: PLoS One. 2008 Jun 18;3(6):e2448. doi: 10.1371/journal.pone.0002448 (PMC2413006; doi:10.1371/journal.pone.0002448)
Supplement: Table S1 — (0.04 MB DOC) [file pone.0002448.s001.doc]

**ONLINE DATA SUPPLEMENT**

**Table S1. Crude association between psoriasis and genetic polymorphisms in PSORS1**

| PSORS1 SNPs  rs# | Genotype | Non psoriasis  n = 1124 | Psoriasis, all  n = 96 | Early-onset psoriasis  n=51 | OR [95% CI] associated with psoriasis | p value | OR [95% CI] associated with early-onset psoriasis | p value |
| --- | --- | --- | --- | --- | --- | --- | --- | --- |
| rs3131000 | GG | 35.1 | 26.0 | 23.5 | 1.00 |  | 1.00 |  |
|  | GC | 50.3 | 50.0 | 52.9 | 1.35 [0.80 ; 2.27] | 0.26 | 1.53 [0.75 ; 3.13] | 0.24 |
|  | CC | 14.6 | 24.0 | 23.5 | 2.23 [1.22 ; 4.08] | 0.009 | 2.31 [1.04 ; 5.12] | 0.04 |
| rs3130559 | CC | 60.6 | 46.9 | 52.9 | 1.00 |  | 1.00 |  |
|  | CT | 35.7 | 44.8 | 41.2 | 1.61 [1.03 ; 2.52] | 0.04 | 1.33 [0.74 ; 2.39] | 0.34 |
|  | TT | 3.7 | 8.3 | 5.9 | 2.96 [1.33 ; 6.57] | 0.008 | 1.85 [0.55 ; 6.22] | 0.32 |
