# Supplementary material for: Replication of Association between ADAM33 Polymorphisms and Psoriasis
Source: PLoS One. 2008 Jun 18;3(6):e2448. doi: 10.1371/journal.pone.0002448 (PMC2413006; doi:10.1371/journal.pone.0002448)
Supplement: Table S2 — (0.04 MB DOC) [file pone.0002448.s002.doc]

**ONLINE DATA SUPPLEMENT**

**Table S2. Pairwise linkage disequilibrium among ADAM33 SNPs (D’ coefficient computed in unrelated individuals: founders and controls, n=1,139)**

|  | rs6084432 | rs512625 | rs677044 | rs543749 | rs628977 | rs2787095 | rs554743 | rs2853215 | rs4815596 |
| --- | --- | --- | --- | --- | --- | --- | --- | --- | --- |
| rs6084432 | 1 | 0.69 | 1.0 | 1.0 | 0.63 | 0.89 | 0.23 | 0.5 | 0.06 |
| rs512625 |  | 1 | 0.41 | 0.91 | 0.34 | 0.5 | 0.33 | 0.22 | 0.42 |
| rs677044 |  |  | 1 | 0.02 | 0.78 | 0.32 | 0.37 | 0.51 | 0.32 |
| rs543749 |  |  |  | 1 | 1.0 | 0.25 | 0.06 | 0.33 | 0.14 |
| rs628977 |  |  |  |  | 1 | 0.26 | 0.27 | 0.38 | 0.19 |
| rs2787095 |  |  |  |  |  | 1 | 0.28 | 0.31 | 0.22 |
| rs554743 |  |  |  |  |  |  | 1 | 0.94 | 0.8 |
| rs2853215 |  |  |  |  |  |  |  | 1 | 1.0 |
| rs4815596 |  |  |  |  |  |  |  |  | 1 |
